# Age‐specific breast cancer incidence by subtype, TNM stage and screening status in Sweden 2008–2019 estimated with multiple imputation

**DOI:** 10.1002/ijc.70355

**Published:** 2026-02-12

**Authors:** Leo Gkekos, Katrín Ásta Gunnarsdóttir, Keith Humphreys, Irma Fredriksson, Anna L. V. Johansson

**Affiliations:** ^1^ Department of Medical Epidemiology and Biostatistics Karolinska Institutet Stockholm Sweden; ^2^ Regional Cancer Center West, Department of Data management and Analysis Region Västra Götaland Gothenburg Sweden; ^3^ Department of Molecular Medicine and Surgery Karolinska Institutet Stockholm Sweden; ^4^ Department of Breast, Endocrine Tumors and Sarcoma Karolinska University Hospital Stockholm Sweden; ^5^ Cancer Registry of Norway Norwegian Institute of Public Health Oslo Norway

**Keywords:** breast cancer, incidence, screening, staging, subtypes

## Abstract

Breast cancer incidence in women increases with age, but less is known about which subtypes contribute the most at different ages. We describe age‐specific breast cancer incidence rates in Sweden by subtype, TNM stage and screening status. Population‐based data were retrieved from the Swedish National Quality Register for Breast Cancer on 89,322 invasive breast cancer cases diagnosed 2008–2019 in women ≥18 years. Breast cancer subtypes were defined by estrogen and progesterone receptors, human epidermal growth factor receptor 2 (HER2), and grade. Poisson regression with multiple imputation estimated proportions and incidence rates. In women <40 years, the breast cancer incidence rates were low with luminal HER2, HER2 positive and triple‐negative breast cancer (TNBC) accounting for 57% of tumors. During screening ages (40–74 years), luminal A‐like tumors accounted for 59% and their incidence rate increased by age. Luminal A‐like tumors were more often screen‐detected (70%) compared to HER2 positive (45%) and TNBC (42%). At age 75 years, the incidence rates declined temporarily for all subtypes, and remained mostly stable in older ages, although the luminal A‐like rates did not reach the same levels as 40–74 years. In the screening ages, stage I incidence rates were increased, especially for the luminal A‐like subtype, while in post‐screening ages (≥75 years), stage II incidence rates were highest, especially for luminal B‐like, luminal HER2 and TNBC subtypes. Age‐specific incidence patterns vary by subtype and are all influenced by screening. Future diagnostic and preventive interventions should consider these age‐specific patterns.

## What's New?

Breast cancer incidence in women increases with age, but which subtypes contribute the most at different ages remains unclear. Incidence patterns of breast cancer subtypes are typically under‐reported in population‐based studies due to a high proportion of cases lacking subtype information. Here, the authors used a nationwide clinical breast cancer registry combined with multiple imputation methods to describe age‐specific subtype incidences in a nationwide setting with screening. Subtype incidences varied substantially by age, likely reflecting non‐identical risk factors, growth patterns, and screening detectability, with different patterns during screening ages. These findings are relevant for preventative healthcare interventions and international comparisons.

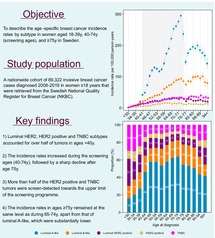

**Abbreviations**
CI: confidence interval
ER: estrogen receptor
HER2: human epidermal growth factor receptor 2
IHC: immunohistochemistry
IR: incidence rates
IRR: incidence rate ratio
MAR: missing at random
MICE: multiple imputation with chained equations
MNAR: missing‐not‐at‐random
NKBC: Swedish National Quality Register for Breast Cancer
PR: progesterone receptor
TNBC: triple‐negative breast cancer
TNM: tumor size, node status, distant metastasis

## 1 INTRODUCTION

The molecular breast cancer subtypes from the early 2000s are routinely approximated by surrogate subtyping based on the estrogen receptor (ER), progesterone receptor (PR), human epidermal growth factor receptor 2 (HER2), grade and/or Ki‐67.^1^ Young women are more often diagnosed with HER2 positive and triple‐negative breast cancer (TNBC) with poorer prognosis compared to luminal subtypes.^2^ In post‐menopausal women, luminal A‐like or luminal B‐like breast cancer subtypes account for a higher proportion of cases. These age‐specific subtype differences likely depend on different (dominant) biological risk mechanisms and risk factors.

Overall, invasive breast cancer incidence rates in women increase steadily with age with a temporary leveling‐off around menopause that increases again in older ages.^3^, ^4^ Population‐based screening contributes to higher incidence rates in screening‐aged women and lower incidence rates in post‐screening ages, not only of invasive breast cancer but also of ductal carcinoma in situ. Screening also changes the subtype distribution of invasive tumors across age, as it generally detects a higher proportion of the slow growing ER+ tumors. Yet, screening will also lead to earlier detection of highly proliferating tumors, such as ER− tumors and especially TNBC, which is important due to their poorer prognosis.^5^, ^6^

Although incidence trends by subtypes have been reported previously, these studies were limited to certain age groups or broad subtypes. Shoemaker et al.^7^ described subtype incidences in ages 20–49 years based on data from The Surveillance, Epidemiology, and End Results (SEER) program, while Mesa‐Eguiagaray et al.^8^ defined surrogate subtypes based on ER/HER2 in Scottish women with breast cancer. A challenge when estimating subtype incidences based on register data is cases with unknown subtype, leading to underestimation of the true rates. Anderson et al.^9^ and Mesa‐Eguaigaray et al.,^8^ both used a simple imputation approach that potentially does not capture the complexity of the missing data.

In Sweden, population‐based screening is offered to all women aged 40–74 years, with an interval of 18–24 months.^10^ In this setting, we aimed to describe the age‐specific breast cancer incidence rates by subtype in women aged <40, 40–74, and ≥75 years. We further describe the effect of screening on subtypes incidence rates in women aged 40–74 years, as well as by stage. To account for unknown subtype, we applied multiple imputation with chained equations (MICE) to ensure that the subtype incidence rates sum to the total breast cancer rate.

## 2 MATERIALS AND METHODS

### 2.1 Study population

This population‐based cohort study was based on Breast Cancer Data Base Sweden (BCBaSe 3.0), a nationwide research database including individuals diagnosed with breast cancer in Sweden 2008–2019. BCBaSe 3.0 includes individual‐level record linkages between the Swedish National Quality Register for Breast Cancer (NKBC) and demographic and national healthcare registers held by the National Board of Health and Welfare and Statistics Sweden.^11^ NKBC records have detailed clinical information on essentially all Swedish residents diagnosed with breast cancer since 2008, with high completeness (>99%).^12^

The study cohort included all cases of primary invasive breast cancer (International Classification of Diseases version 10: C50) registered in BCBaSe 3.0 in women aged ≥18 years during 2008–2019. Up to two occurrences of invasive breast cancer were possible for each woman: one for each breast, and regardless of previous history of cancer. The Swedish female population at risk was extracted by age and year from publicly available data from Statistics Sweden.

Surrogate subtypes were defined based on the immunohistochemistry (IHC) evaluation of ER, PR, HER2, and grade according to the Swedish treatment guidelines. ER and PR were considered positive when ≥10% of the tumor cells stained positive. HER2 was deemed positive if IHC 3+ or 2+ with gene amplification according to in situ hybridization.

Surrogate subtypes were: luminal A‐like (ER+/PR+/HER2−/grades I–II, ER+/PR–/HER2−/grade I or ER–/PR+/HER2−/grades I–II), luminal B‐like (ER+/PR+/HER2−/grade III, ER+/PR–/HER2−/grades II–III or ER–/PR+/HER2−/grade III), luminal HER2 (ER+/PR+/HER2+/grades I–III, ER+/PR–/HER2+/grades I–III or ER–/PR+/HER2+/grades I–III), HER2 positive (ER–/PR–/HER2+/grades I–III), and TNBC (ER–/PR–/HER2−/grades I–III). Data on ER status were missing in 4.1% of tumors, PR in 4.2%, HER2 in 7.9%, and grade for 16.4%, and consequently, 15.5% of the tumors could not be subtyped.

TNM (tumor size, node status, distant metastasis) stage was based on clinical and pathological information according to the Union for International Cancer Control, 8th edition.^13^ If primary surgery was given, pathological T stage was preferred over clinical T stage. Pathological N stage was preferred over the clinical N stage, except for when node negative after neoadjuvant treatment. Information on T stage was missing for 0.3% of tumors, N stage for 0.7%, while M stage was complete. The combined TNM stage was missing in 0.5% of cases.

Information on mode of detection of tumors is recorded in NKBC, and cases were classified as screen‐detected within the population‐based screening program or symptomatically detected outside the program.

### 2.2 Statistical analysis

Incidence rates (IRs) per 100,000 women were calculated as the number of cases divided by population counts in 1‐year age and 1‐year calendar period categories. IRs for each subtype were estimated and were further broken down by screening status and stage. Poisson regression models were used to estimate incidence rate ratios (IRRs) with 95% confidence intervals (CI) and included either age at diagnosis in 2‐year (reference level: 60–61 years) or 5‐year intervals (reference level: 60–64 years) or year of diagnosis in 1‐year intervals (reference level: 2008) as covariates.

Due to missing values on ER, PR, HER2, grade and stage, we performed MICE for the Poisson regression analyses to avoid a loss in efficiency and minimize bias.^14^, ^15^ We generated 30 imputed datasets with 20 iterations each by replacing missing values with simulated values from imputation models based on all predictor and auxiliary variables (Supporting Information Methods). The distributions of the imputed values were compared visually to observed distributions. Poisson regression was performed on each imputed dataset and the pooled parameter estimates were obtained using Rubin's rules, which can be interpreted under the assumption of missing at random (MAR). Estimated counts, proportions and incidence rates were obtained from the model parameters.

We used Stata 18/BE for all analyses, and Stata mi package for the multiple imputation (StataCorp. 2023. Stata Statistical Software: Release 18. College Station, TX: StataCorp LLC).

## 3 RESULTS

During 2008–2019, 89,332 invasive breast cancer tumors were identified in the cohort. Among women with known subtype (84.5%), 56.5% were luminal A‐like, 19.2% luminal B‐like, 9.8% luminal HER2, 4.6% HER2 positive, and 9.9% TNBC (Table S1). The percentage of missing subtype decreased from 21.1% (2008) to 14.5% (2019). Luminal A‐like tumors were most commonly stage I (61.9%), whereas stage II tumors were most common in all other subtypes (Table S2). HER2 positive, luminal HER2, and TNBC tumors were more advanced at diagnosis (stage III‐IV: 25%, 18%, and 16%, respectively).

During 2008–2019, the IRs of all subtypes remained stable (Figure S1). The total breast cancer IR increased by age with a brief plateau around age 50–55 years, followed by a sharp decline at the end of screening (74 years), and a second decline starting at ages 88–89 years (Figure S2).

### 3.1 Age‐specific incidence rates and proportions by subtype

**FIGURE 1:**
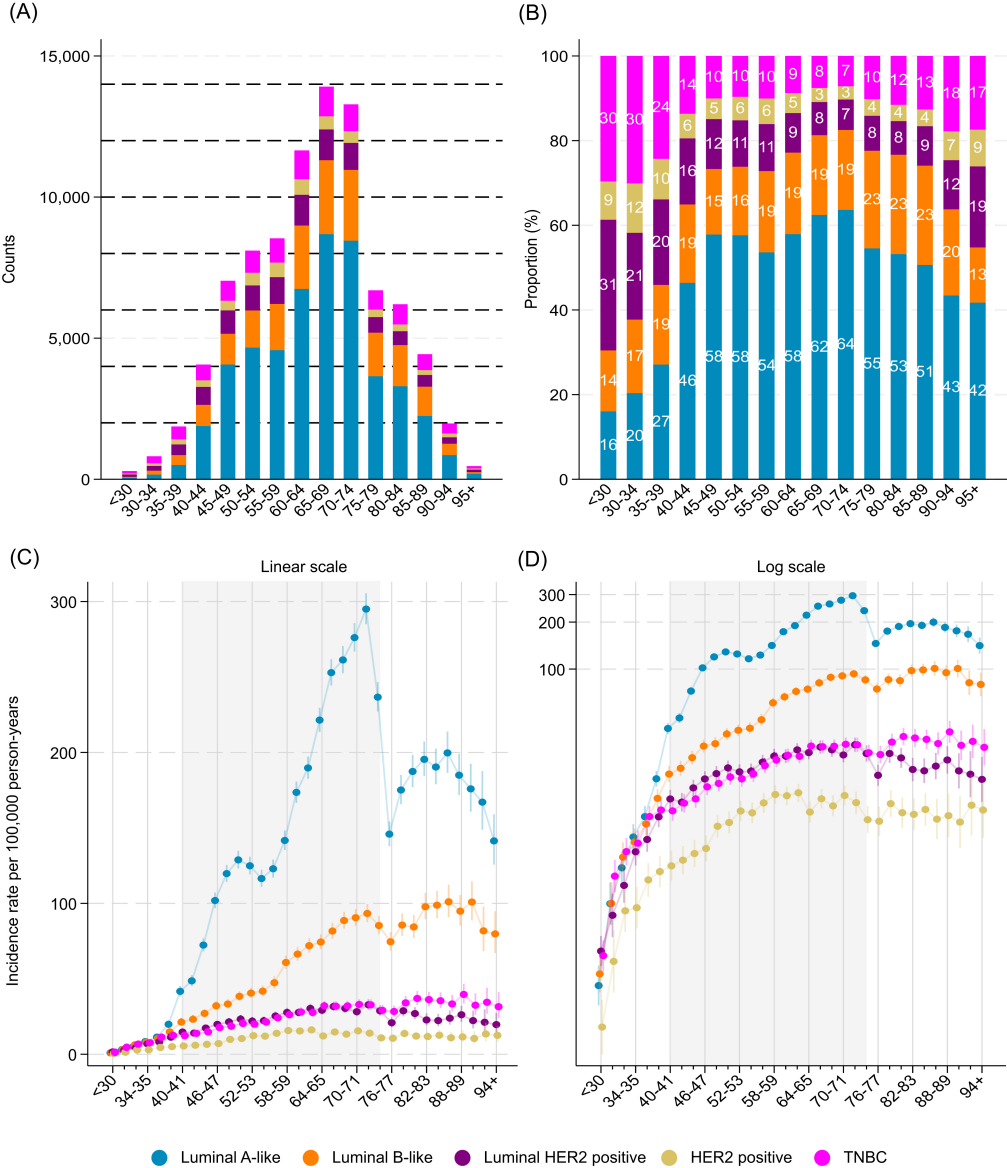
Estimated counts (A), proportions (B) and incidence rates (C: linear scale, D: logarithmic scale) of breast cancer subtypes by age at diagnosis in Sweden 2008–2019—based on Multiple Imputed data. Shaded area represents screening ages 40–74 years.

Among women aged <40 years, 24% had luminal A‐like, 18% luminal B‐like, 21% luminal HER2, 10% HER2 positive and 26% TNBC tumors based on estimated proportions from the imputation analysis (Figure 1A,B; Figure S3). The estimated proportion of luminal HER2 tumors was highest in women <30 years (31%). In contrast, among screening ages (40–74 years), 59% of tumors were luminal A‐like, increasing from 46% (40–44 years) to 64% (70–74 years), while the proportion of luminal B‐like tumors was stable over age and the proportions of luminal HER2, HER2 positive and TNBC subtypes declined. In post‐screening ages (≥75 years), the TNBC proportion increased by age (90–94 years: 18%), while the luminal A‐like proportion decreased (90–94 years: 42%).

The estimated IRs were very low in women <40 years and increased up to age 74 years across all subtypes, with a temporary plateau around 50–55 years (Figure 1C,D). In ages below 38 years, the five subtype IRs were fairly similar, with the exception of a lower IR for HER2 positive subtype (Figure S4). From ages 38–39 years onwards the luminal A‐like and luminal B‐like IRs were the highest. In ages 40–74 years, the luminal A‐like IR increased until end of screening (74 years), with the aforementioned plateau at 50–55 years, which was less noticeable for the other subtypes (Figure 1C,D). The luminal B‐like IR followed the same pattern as the luminal A‐like yet was lower. The luminal HER2 and TNBC IRs remained similar across ages 40–74 years, although with a slightly higher IR of luminal HER2 during ages 40–53 years. In contrast, the HER2 positive IR increased substantially during ages 40–59 years after which it leveled off. At the end of screening (74 years), the IRs of luminal A‐like and luminal B‐like subtypes temporarily declined sharply. The luminal A‐like IR after age 75 years rebounded yet remained lower than during screening ages, while the luminal B‐like IR rebounded to a similar level as during screening ages. Also, the IRs of luminal‐HER2, HER2 positive and TNBC subtypes declined at end of screening, but returned to a higher level faster than the other subtypes. The TNBC IR continued to increase and peaked at ages 88–89 years, while the luminal HER2 IR declined after age 75 years.

**FIGURE 2:**
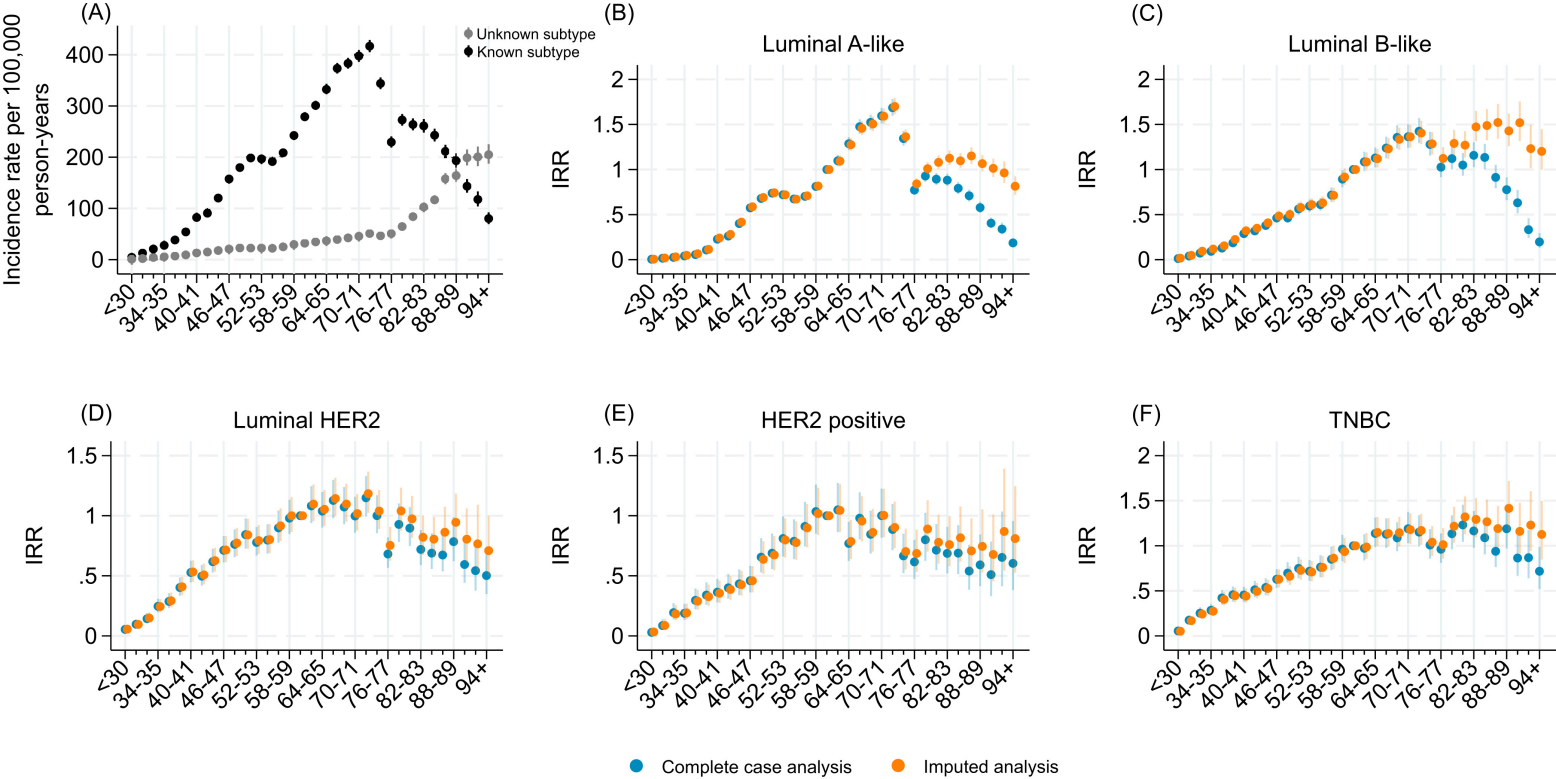
Breast cancer incidence rates of known and unknown subtype by age at diagnosis (A) and comparison of incidence rate ratios (IRRs) estimated by the imputed and complete case analyses (B: luminal A‐like, C: luminal B‐like, D: luminal HER2, E: HER positive F: triple negative breast cancer/TNBC).

Importantly, the proportion of unknown subtype increased sharply among the oldest (>80 years) (Figure 2A). Comparing IRRs from the complete case and imputation analyses indicated that the imputation model added information similarly up until age 75 years. From age 75 years, higher IRRs were estimated for all subtypes by the imputed analysis with a larger proportion imputed as luminal A‐like and luminal B‐like compared to the other subtypes (Figure 2B–F).

### 3.2 Incidence rates by subtype and the effect of screening

**FIGURE 3:**
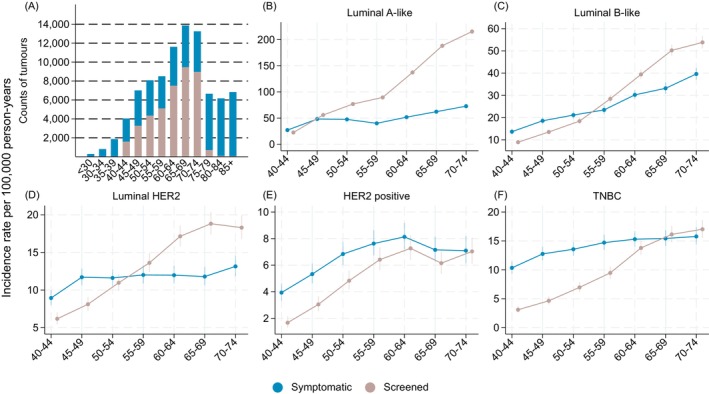
Estimated counts (A) and incidence rates (B: luminal A‐like, C: luminal B‐like, D: luminal HER2, E: HER positive F: triple negative breast cancer/TNBC) of symptomatic and screen‐detected breast cancer tumors by age at diagnosis and subtype in Sweden 2008–2019—based on Multiple Imputed data.

In ages 40–74 years, the estimated numbers and IRs of screen‐detected tumors increased with age across all subtypes (Figure 3A–F). However, luminal A‐like tumors were more often screen‐detected from age 50 years and onwards, while the IRs of screen‐detected luminal B‐like and luminal HER2 surpassed those of symptomatic at ages 55–59 years, and at ages 70–74 for HER2 positive and TNBC subtypes.

**FIGURE 4:**
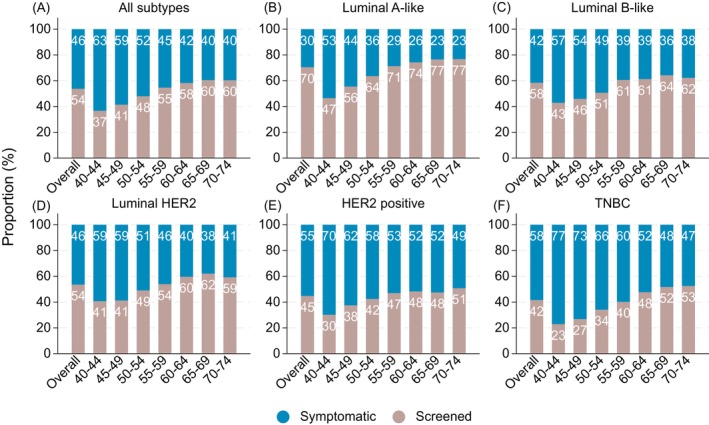
Estimated proportions of symptomatic and screen‐detected breast cancer tumors by age at diagnosis and subtype in screening ages 40–74 in Sweden 2008–2019—based on Multiple Imputed data. (A: all subtypes, B: luminal A‐like, C: luminal B‐like, D: luminal HER2, E: HER positive F: triple negative breast cancer/TNBC).

The estimated proportion of screen‐detected tumors increased by age for all subtypes (Figure 4A). For luminal A‐like, this proportion increased from 47% (40–44 years) to 77% (70–74 years) (panel B), for luminal B‐like from 43% to 62% (panel C), for luminal HER2 from 41% to 59% (panel D), for HER2 positive from 30% to 51% (panel E), and for TNBC from 23% to 53% (panel F).

### 3.3 Age‐specific incidence rates by stage and subtype

**FIGURE 5:**
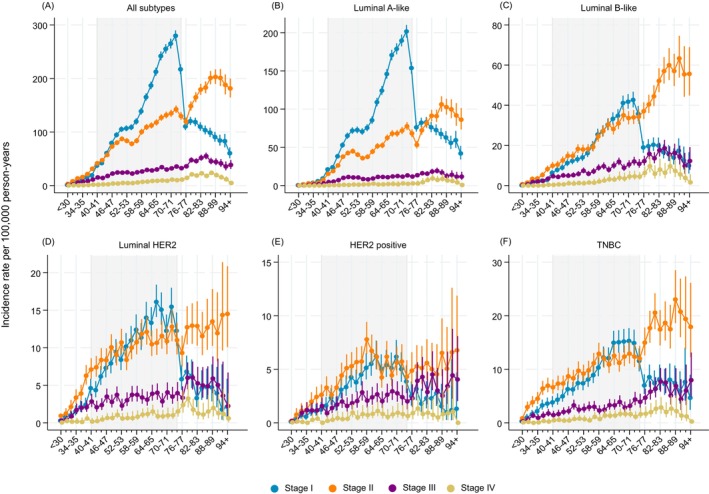
Estimated age‐specific breast cancer incidence by stage and subtype in Sweden 2008–2019—based on imputed data. (A: all subtypes, B: luminal A‐like, C: luminal B‐like, D: luminal HER2, E: HER positive F: triple negative breast cancer/TNBC).

Among ages <40 years, the stage II IR was the highest, both overall and for luminal B‐like, luminal HER2, HER2 positive and TNBC especially (Figure 5A,C–F). For the luminal A‐like subtype, the stage I IR was the highest during screening ages with a peak at ages 70–74 years, declining sharply afterwards (panel B). In contrast, the stage II IR increased moderately until 75 years and continued to increase after 75 years. Both stage III and stage IV IRs increased slightly during screening ages, and also between ages 75 and 85 years, after which they declined. For the luminal B‐like subtype, the stage I and II IRs were similar and increased during screening ages, after which stage I IR declined while stage II IR continued to increase (panel C). Stage III and IV IRs increased modestly during ages 40–74 years followed by a modest decrease from age 80 years. The luminal HER2 IRs followed a similar pattern as luminal B‐like (panel D). For HER2 positive subtype, stage II IRs were the highest throughout all ages, while stage I IR declined sharply in ages ≥75 years (panel E). For TNBC, the stage‐specific IRs were similar to luminal B‐like and luminal HER2 subtypes during screening ages, while stage II rates increased in post‐screening ages ≥75 years, and all other stage IRs declined (panel F). When comparing the complete case and imputation analyses, a larger proportion of tumors in the older age groups was imputed as luminal A‐like and luminal B‐like compared to the other subtypes, as expected (Figure S5). Finally, estimated stage distributions by subtype for all ages are presented in Figure S6.

## 4 DISCUSSION

In this nationwide study, breast cancer subtypes exhibited distinct age‐varying incidence rates likely reflecting a combination of risk factor effects, different growth patterns, and screening detectability. In pre‐screening ages (<40 years), luminal HER2, HER2 positive, and TNBC subtypes accounted for over half of tumors and more than two thirds in ages <30 years, indicating a general pattern of more sinister tumors in comparison to middle‐aged women. In the youngest, luminal HER2 tumors were more common than HER2 positive. During the screening ages (40–74 years), the rates increased in particular for luminal A‐like tumors, which was followed by a sharp decline at age 75 years. The proportion of screen‐detected tumors increased over age, also among non‐luminal subtypes, where more than half of HER2 positive and TNBC tumors were detected by screening in older ages. In post‐screening ages (≥75 years), luminal A‐like rates were substantially lower than during ages 65–74 years and declined after age 85 years, while rates of the other subtypes remained at the same level as during 65–74 years. The stage distribution of pre‐screening and post‐screening ages was similar, while the stage I rates were higher during screening ages, in particular for luminal A‐like tumors.

### 4.1 Age‐specific incidence rates and proportions by subtype

Corroborating our findings, previous studies have reported higher proportions of HER2 positive and TNBC in ages <40 years compared to middle‐aged women, and subsequently a lower survival and a higher risk of recurrence at any clinical stage.^2^, ^16^, ^17^, ^18^, ^19^

During screening ages, luminal tumors are the most common subtypes,^7^, ^8^, ^9^, ^20^, ^21^ with increasing rates of ER+ tumors by age in the USA, Denmark, and Scotland. In these countries, decreasing rates of ER− tumors were observed during screening ages, which is not consistent with our results.^8^, ^22^, ^23^ We observed a plateau in luminal A‐like rates at ages 50–59 years, which was more prominent than that reported in the USA by Howlader et al.,^20^ and reminiscent of the plateau at menopause frequently observed in unscreened populations.^21^, ^24^ According to an evaluation of the Swedish population‐based mammography screening program, 81% of invited women participated in screening in Sweden 2017–2018, with similar participation across ages (40–54 years: 79%, 55–74 years: 82%), meeting the World Health Organization target level of 70%.^25^, ^26^

As expected, we observed declining rates after cessation of screening, which were pronounced for luminal A‐like and less for HER2 positive and TNBC subtypes. This is consistent with lead times being longer for slow‐growing tumors.^27^, ^28^ Mesa‐Eguiagaray et al.^8^ and Howlader et al.^20^ demonstrated similarly decreasing rates at post‐screening ages. In the oldest ages, this trend could also be the result of under‐diagnosis due to changed health‐seeking behavior, comorbidities and related decisions not to operate. Only women with low comorbidity and tumors not appropriate for primary endocrine therapy will undergo surgery and be investigated for subtype, while frail women with hormone receptor positive tumors at cytology will more often only receive endocrine therapy without surgical specimen for subtyping biomarker analysis. Thus, as expected, the imputation model gave higher relative increases of luminal HER, HER2 positive and TNBC in older women. Contrary to our finding, Jenkins et al^29^ showed an increasing proportion of luminal tumors with age, yet they considered ages 70–93 years as one group.

### 4.2 The effect of screening on the age‐specific incidence rates and proportions by subtype

Mammographic screening has previously been suggested to favor the detection of luminal A‐like and B‐like tumors.^30^, ^31^ We found the luminal subtypes more often to be screen‐detected than HER2 positive and TNBC, yet the proportion of screen‐detected tumors increased steadily by age across all subtypes, reflecting the decreasing breast density over age and a higher screening participation in older women. Importantly, the rates of screen‐detected HER2 positive and TNBC subtypes increased with age, which should translate to less intense treatment and better prognosis.

### 4.3 Age‐specific incidence rates by stage and subtype

We found that tumors in ages <40 years were most often diagnosed at stage II except for luminal A‐like tumors, which were primarily stage I. Young women are typically diagnosed at later stages, which contributes to lower survival rates.^18^ Additionally, a higher proportion of early‐onset breast cancer is hereditary,^32^ and exhibit increased expression of markers associated with proliferation, stem cells, and resistance to endocrine therapy compared with tumors in older women.^33^

Increased rates of stage I disease among luminal A‐like tumors during screening ages, and equally high rates of stages I and II among luminal B‐like and luminal HER2 have also been previously reported.^34^ Howlader et al. reported higher stage III rates among HER2 positive tumors.^20^ This is expected given the slow growth of luminal A‐like tumors and the early detection due to screening, yet it is important to quantify these effects both in absolute and relative numbers

Lastly, in the post‐screening ages (≥75 years), most breast cancers were diagnosed at stage II. Opportunistic screening in ages 75–79 years could potentially explain that stage I luminal A‐like tumors were more common than stage II in these ages. Although older women tend to have more advanced tumors,^35^ the decreasing rates of stage III and IV in older women (≥90 years) in our cohort could also reflect a certain degree of understaging due to comorbidities and frailty.

## 5 STRENGTHS AND LIMITATIONS

The major strength of our study includes the high‐quality and >99% complete population‐based data of NKBC.^12^ To account for the non‐negligible proportion of missing data on breast cancer subtype, we utilized MICE to avoid underestimation of subtype incidences. The validity of MICE depends on the quality of the imputation model; hence, we included a thorough list of predictors in the model. However, we cannot rule out that some model misspecification may have influenced the final results, in particular in older ages. MICE also relies on the MAR assumption, and despite the extensive imputation model, we cannot exclude that some variables were missing‐not‐at‐random (MNAR), that is, missingness depending on unobserved variables. However, removing either the earlier calendar period (2008–2014) or certain auxiliary variables did not influence the results. A final limitation is the non‐perfect alignment between molecular and surrogate subtypes, and therefore the incidence rates are not directly transferable. However, during the studied period, molecular subtyping was rarely used in routine treatment decisions.

## 6 CONCLUSION

In conclusion, breast cancer subtypes exhibit incidence patterns which are highly age‐dependent and impacted by screening. These findings can inform clinical professions, healthcare decision makers, and researchers by providing insights into the subtype‐specific breast cancer burden at different ages in a screened population. This is important for healthcare resource allocation, planning of detailed clinical studies and for international comparisons. These incidence patterns also add important new knowledge about the underlying biology of breast cancer, as they suggest differential underlying risk factors of breast cancer subtypes, which should be taken into account when designing diagnostic and preventative interventions. More research is needed to confirm and understand the shifts of breast cancer subtypes in younger women and in post‐screening ages.

## AUTHOR CONTRIBUTIONS

**Leo Gkekos:** Writing – original draft; methodology; visualization; data curation; formal analysis; conceptualization. **Katrín Ásta Gunnarsdóttir:** Writing – review and editing; data curation. **Keith Humphreys:** Conceptualization; writing – review and editing; methodology; supervision. **Irma Fredriksson:** Supervision; writing – review and editing; conceptualization. **Anna L. V. Johansson:** Supervision; funding acquisition; conceptualization; methodology; writing – review and editing.

## FUNDING INFORMATION

This work was supported by the Swedish Cancer Society (grant numbers 22 2044 Pj, 24 0790 SIA, Anna Johansson); the Swedish Research Council (grant number 2021‐01657, Anna Johansson). Irma Fredriksson was funded through the Erik och Majje Näsström donation to Karolinska Institutet. Keith Humphreys was funded by the Swedish Research Council (grant number 2023‐02063).

## CONFLICT OF INTEREST STATEMENT

Irma Fredriksson has received an institutional research grant from MSD, unrelated to the current work, initiated and published in accordance with a Master Collaboration Agreement between Karolinska Institutet and the company. She is Chairman of the Swedish National Quality Register for Breast Cancer, Board Member of the Swedish Breast cancer Group, SewBCG, and Board Member of the Percy Falck Research Foundation for Breast and Prostatic Cancer. The other authors declare no conflict of interests.

## ETHICS STATEMENT

This work has received approval by the Swedish Ethical Review Authority (responsible researcher BCBaSe3.0: Irma Fredriksson, DNR 2019‐02610, and amendments 2020‐00886, 2020‐06302, 2022‐01020, 2022‐01089‐02, responsible researcher for amendment: Anna L V Johansson, DNR 2023‐01511‐02).

## Supporting information


**Data S1.** Supporting information.

## Data Availability

The data supporting this article was obtained from three Swedish register holders (Regional Cancer Center, Stockholm‐Gotland, Sweden; The Swedish National Board of Health and Welfare, Sweden; Statistics Sweden) under specific ethical approval by the Swedish Ethical Review Authority. Researchers with appropriate approvals can apply for Swedish health register data from these authorities. Further information is available from the corresponding author upon request.
