# Supplementary material for: Age‐specific breast cancer incidence by subtype, TNM stage and screening status in Sweden 2008–2019 estimated with multiple imputation
Source: Int J Cancer. 2026 Feb 12;159(1):101–10. doi: 10.1002/ijc.70355 (PMC13140068; doi:10.1002/ijc.70355)
Supplement: Supplementary file 1 — Data S1. Supporting information. [file IJC-159-101-s001.pdf]

Age-specific breast cancer incidence by subtype, TNM stage and screening status in Sweden 2008-2019 estimated with multiple imputation

Leo Gkekos, Katrín Ásta Gunnarsdóttir, Keith Humphreys, Irma Fredriksson, Anna L.V. Johansson

Contents

Supplemental methods 2

Supplemental table S1 5

Supplemental table S2 7

Supplemental figure S1 9

Supplemental figure S2 10

Supplemental figure S3 11

Supplemental figure S4 12

Supplemental figure S5 13

Supplemental figure S6 14

References 15

Supplemental methods

Details of multiple imputation

We applied multiple imputation with chained equations (MICE) to account for missing information in important covariates (1). Multiple imputation is a two-step procedure involving the imputation step generating imputed datasets, and an analysis step where the analytical model is applied to each imputed dataset and the results are pooled together to combined estimates using Rubin's rules.

Preparation for imputation

Table S1 lists the included variables in the imputation model (step 1) and the analytical model (step 2). Several variables were complete, while a few included missing data. The size of the dataset in the imputation analysis was N=89,332.

The variables that were imputed include: ER, PR, HER2, surgery, chemotherapy, radiotherapy, endocrine therapy, targeted therapy, grade, nodal involvement N, educational level at diagnosis, T stage, and whether the tumour was screen-detected or not. Of the variables included in the imputation models, all but two were auxiliary variables that were not included in the analytical models; the two being age at diagnosis and year of diagnosis. In addition, we included the event indicators for breast cancer specific survival (BCSS) and all-cause overall survival (OS), as well as the Nelson-Aalen estimators for BCSS and OS, as suggested by Falcaro and colleagues (2). Survival was estimated with follow-up until the end of 2019. Assumption before imputation: No distant metastasis was assumed if M stage was unknown.

Overview of variables included in the imputation model and analytical model.

	**Missing among N=89,332 (proportion missing)**	**Deterministic predictions before imputation**	**Type in imputation N=89,332**	**Imputation model** X=included	**Analytical Poisson models** X=included
Age at diagnosis	Complete		Complete	X	X
Year of diagnosis	Complete		Complete	X	X
Region	Complete		Auxiliary	X	
Country of birth	Complete		Auxiliary	X	
Educational level at diagnosis	1,055/89,332 (1.18%)		Auxiliary	X	

Screening status	386/89,332 (0.43%)		Auxiliary	X	
T	251/89,332 (0.28%) missing		Auxiliary	X	
N	606/89,332 (0.68%)		Auxiliary	X	
M	Complete	X	Complete/Auxiliary	X	
Stage	465/89,332 (0.52%)				X
Grade	14,610/89,332 (16.35%)		Auxiliary	X	
ER	3,657/89,332 (4.09%)		Auxiliary	X	
PR	3,778/89,332 (4.23%)		Auxiliary	X	
HER2	7,049/89,332 (7.89%)		Auxiliary	X	
Subtype	13,857/89,332 (15.51%)				X
Surgery	7,046/89,332 (7.89%)		Auxiliary	X	
Chemotherapy	17,818/89,332 (19.95%)		Auxiliary	X	
Radiotherapy	17,941/89,332 (20.08%)		Auxiliary	X	
Endocrine therapy	17,535/89,332 (19.63%)		Auxiliary	X	
Targeted therapy	17,992/89,332 (20.14%)		Auxiliary	X	
dead_BC_ind, dead_ind, cumhaz_BCSS, cumhaz_OS	Complete		Auxiliary	X	

dead_BC_ind=event indicator for BCSS
dead_ind=event indicator for OS
cumhaz_BCSS=Nelson-Aalen estimator for BCSS
cumhaz_OS=Nelson-Aalen estimator for OS

Step 1: Imputation model

We used Stata mi package including command mi impute (Stata/BE 17.0). A total of *m*=30 imputed datasets, with 20 iterations, were generated. The number of imputed datasets *m* was chosen to be larger than the highest percentage of missing values among the variables (Targeted therapy=20.14%), according to the rule of thumb by White and colleagues (2011) (1). The M stage variable was deterministically predicted before it was included in the imputation model; all tumours with missing information were considered not to have any distant metastases. In the chained equations, logistic regression models were applied to binary outcome variables (ER, PR, HER2, chemotherapy, radiotherapy, endocrine therapy, targeted therapy, screening status), while ordinal regression was used for categorical outcome variables (grade, nodal involvement N, surgery type, T stage, educational level). The imputation model was stratified by age at diagnosis (using the *by* option in mi estimate command), thus corresponding to including all pairwise interactions of age at diagnosis (<40, 40-44, 45-49, 50-54, 55-59, 60-64, 65-69, 70-74, 75-79, ≥80) with each covariate in the model. The variables included are listed in the table above.

Step 2: Analysis model and pooling

We combined the ER, PR, HER2 and grade into a subtype variable using mi passive: utility in Stata. Some analytical Poisson models included age at diagnosis and some others included year of diagnosis. To estimate pooled IRR's and incidence rates, we manually applied Rubin's rules to the imputed datasets.

Supplemental table S1

Subtype distribution among 89,332 cases of breast cancer by year of diagnosis, age at diagnosis, screening status and stage in Sweden 2008-2019.

	Luminal A-like N (row %) *	Luminal B-like N (row %) *	Luminal HER2 N (row %)*	HER2 positive N (row %) *	TNBC N (row %) *	Missing N (row %)
**Total no. of observations**	42643 (56.5)	14435 (19.2)	7431 (9.8)	3471 (4.6)	7495 (9.9)	13857 (15.5)
**Year of diagnosis**						
2008	2973 (55.8)	1015 (19.1)	502 (9.4)	264 (5.0)	574 (10.8)	1427 (21.1)
2009	3017 (56.1)	1067 (19.8)	476 (8.9)	251 (4.7)	565 (10.5)	1365 (20.2)
2010	3307 (56.8)	1179 (20.2)	536 (9.2)	266 (4.6)	535 (9.2)	1344 (18.8)
2011	3663 (57.2)	1199 (18.7)	663 (10.3)	271 (4.2)	613 (9.6)	1191 (15.7)
2012	3681 (58.5)	1259 (20.0)	563 (8.9)	277 (4.4)	513 (8.2)	1202 (16.0)
2013	3878 (58.4)	1319 (19.8)	607 (9.1)	247 (3.7)	595 (9.0)	832 (11.1)
2014	3871 (57.7)	1232 (18.4)	677 (10.1)	315 (4.7)	614 (9.2)	1024 (13.2)
2015	3731 (56.6)	1211 (18.4)	657 (10.0)	309 (4.7)	679 (10.3)	1021 (13.4)
2016	3575 (56.1)	1173 (18.4)	654 (10.3)	301 (4.7)	669 (10.5)	1008 (13.7)
2017	3647 (55.3)	1276 (19.3)	690 (10.5)	324 (4.9)	660 (10.0)	1154 (14.9)
2018	3549 (54.6)	1244 (19.1)	693 (10.7)	303 (4.7)	709 (10.9)	1126 (14.8)
2019	3751 (54.9)	1261 (18.4)	713 (10.4)	343 (5.0)	769 (11.2)	1163 (14.5)
**Age at diagnosis**						
18-24	8 (21.1)	8 (21.1)	12 (31.6)	3 (7.9)	7 (18.3)	3 (7.3)
25-29	40 (16.5)	31 (12.8)	70 (28.9)	24 (9.9)	77 (31.8)	57 (19.1)
30-34	146 (20.1)	131 (18.0)	153 (21.1)	81 (11.2)	215 (29.6)	153 (17.4)
35-39	501 (28.9)	326 (18.8)	339 (19.6)	160 (9.2)	405 (23.4)	303 (14.9)
40-44	1782 (47.6)	699 (18.7)	557 (14.9)	222 (5.9)	481 (12.9)	592 (13.7)
45-49	3769 (58.2)	969 (15.0)	772 (11.9)	313 (4.8)	648 (10.0)	826 (11.3)
50-54	4172 (57.6)	1198 (16.6)	791 (10.9)	396 (5.5)	680 (9.4)	826 (10.2)
55-59	4154 (53.4)	1496 (19.2)	860 (11.1)	470 (6.0)	801 (10.3)	968 (11.1)
60-64	6302 (58.3)	2121 (19.6)	971 (9.0)	489 (4.5)	925 (8.6)	1214 (10.1)
65-69	7948 (62.9)	2350 (18.6)	973 (7.7)	432 (3.4)	941 (7.4)	1372 (9.8)
70-74	7160 (63.5)	2149 (19.1)	816 (7.2)	344 (3.1)	808 (7.2)	1375 (10.9)
75-79	2838 (53.6)	1229 (23.2)	446 (8.4)	214 (4.0)	569 (10.7)	1223 (18.8)
80-84	2295 (52.8)	1045 (24.0)	347 (8.0)	171 (3.9)	488 (11.2)	1717 (28.3)
85-89	1208 (50.6)	551 (23.1)	223 (9.3)	98 (4.1)	308 (12.9)	1838 (43.5)
90-94	289 (43.7)	124 (18.7)	79 (11.9)	46 (6.9)	124 (18.7)	1111 (62.7)
95+	31 (35.6)	8 (9.2)	22 (25.3)	8 (9.2)	18 (20.7)	279 (76.2)
**Screening detection**						
No	17436 (46.9)	7921 (21.3)	4308 (11.6)	2257 (6.1)	5244 (14.1)	10606 (22.2)
Yes	25085 (65.9)	6470 (17.0)	3101 (8.1)	1204 (3.2)	2219 (5.8)	3101 (7.5)
Missing	122	44	22	10	32	150

No (ages 40-74)	10389 (47.0)	4550 (20.6)	2644 (12.0)	1462 (6.6)	3063 (13.9)	4088 (15.6)
Yes (ages 40-74)	24806 (65.8)	6403 (17.0)	3077 (8.2)	1195 (3.2)	2197 (5.8)	2999 (7.4)
Missing (ages 40-74)	92	29	19	9	24	86
**TNM Stage**						
Stage I	26369 (67.8)	5930 (15.3)	2845 (7.3)	1087 (2.8)	2633 (6.8)	3977 (9.3)
Stage II	13746 (47.8)	6612 (23.0)	3245 (11.3)	1515 (5.3)	3636 (12.6)	5100 (15.1)
Stage III	2466 (34.4)	1853 (25.9)	1115 (15.6)	726 (10.1)	1000 (14.0)	2383 (25.0)
Stage IV	25 (4.2)	22 (3.7)	211 (35.6)	130 (22.0)	204 (34.5)	2037 (77.5)
Missing	37	18	15	13	22	360

* Percentage calculated among breast cancers with known subtype.

Supplemental table S2

Stage distribution among 89,332 cases of breast cancer by year of diagnosis, age at diagnosis, screening status, and subtype in Sweden 2008-2019.

	Stage I<br>N (row %) *	Stage II<br>N (row %) *	Stage III<br>N (row %)*	Stage IV<br>N (row %) *	Missing<br>N (row %)
**Total no. of observations**	42841 (48.2)	33854 (38.1)	9543 (10.7)	2629 (3.0)	465 (0.5)
**Year of diagnosis**					
2008	3156 (47.2)	2471 (36.9)	889 (13.3)	174 (2.6)	65 (1.0)
2009	3049 (45.5)	2618 (39.1)	847 (12.7)	180 (2.7)	47 (0.7)
2010	3372 (47.4)	2691 (37.9)	841 (11.8)	204 (2.9)	59 (0.8)
2011	3584 (47.5)	2892 (38.4)	866 (11.5)	199 (2.6)	59 (0.8)
2012	3677 (49.4)	2663 (35.8)	882 (11.9)	214 (2.9)	59 (0.8)
2013	3635 (48.8)	2827 (38.0)	791 (10.6)	191 (2.6)	34 (0.5)
2014	3760 (48.7)	2932 (38.0)	800 (10.4)	221 (2.9)	20 (0.3)
2015	3788 (50.0)	2804 (37.0)	788 (10.4)	203 (2.7)	25 (0.3)
2016	3578 (48.7)	2831 (38.5)	676 (9.2)	265 (3.6)	30 (0.4)
2017	3756 (48.5)	2961 (38.3)	756 (9.8)	267 (3.4)	11 (0.1)
2018	3664 (48.2)	3000 (39.5)	654 (8.6)	276 (3.6)	30 (0.4)
2019	3822 (47.9)	3164 (39.7)	753 (9.4)	235 (2.9)	26 (0.3)
**Age at diagnosis**					
18-24	9 (22.0)	27 (65.9)	5 (12.1)	0 (0.0)	0 (0.0)
25-29	81 (27.3)	142 (47.8)	60 (20.2)	14 (4.7)	2 (0.7)
30-34	230 (26.2)	448 (51.0)	178 (20.3)	22 (2.5)	1 (0.1)
35-39	629 (31.1)	990 (48.9)	358 (17.7)	48 (2.4)	9 (0.4)
40-44	1791 (41.5)	1845 (42.7)	602 (13.9)	79 (1.8)	16 (0.4)
45-49	3350 (46.0)	2910 (40.0)	880 (12.1)	144 (2.0)	13 (0.2)
50-54	3923 (48.8)	3060 (38.0)	885 (11.0)	177 (2.2)	18 (0.2)
55-59	4484 (51.4)	3120 (35.8)	900 (10.3)	221 (2.5)	24 (0.3)
60-64	6661 (55.5)	4019 (33.5)	1041 (8.7)	283 (2.4)	18 (0.1)
65-69	8306 (59.4)	4285 (30.6)	1072 (7.7)	322 (2.3)	31 (0.2)
70-74	7473 (59.2)	3895 (30.9)	954 (7.6)	293 (2.3)	37 (0.3)
75-79	2447 (37.8)	2798 (43.2)	862 (13.3)	367 (5.7)	45 (0.7)
80-84	1866 (31.2)	2882 (48.1)	893 (14.9)	346 (5.8)	76 (1.3)
85-89	1098 (26.6)	2228 (53.9)	564 (13.6)	242 (5.9)	94 (2.2)
90-94	412 (24.1)	991 (57.9)	241 (14.1)	68 (4.0)	61 (3.4)
95+	81 (23.4)	214 (61.8)	48 (13.9)	3 (0.9)	20 (5.5)
**Screening detection**					
No	16019 (33.9)	21850 (46.2)	7055 (14.9)	2361 (5.0)	396 (0.8)
Yes	26689 (64.7)	11889 (28.8)	2447 (5.9)	200 (0.5)	40 (0.1)
Missing	133	115	41	68	29

No (ages 40-74)	9534 (36.6)	11361 (43.6)	3910 (15.0)	1276 (4.9)	115 (0.4)
Yes (ages 40-74)	26352 (64.8)	11703 (28.8)	2395 (5.9)	193 (0.5)	34 (0.1)
Missing (ages 40-74)	102	70	29	50	8
**Subtype**					
Luminal A-like	26369 (61.9)	13746 (32.3)	2466 (5.8)	25 (0.1)	37 (0.1)
Luminal B-like	5930 (41.1)	6612 (45.9)	1853 (12.9)	22 (0.2)	18 (0.1)
Luminal HER2	2845 (38.4)	3245 (43.8)	1115 (15.0)	211 (2.8)	15 (0.2)
HER2 positive	1087 (31.4)	1515 (43.8)	726 (21.0)	130 (3.8)	13 (0.4)
TNBC	2633 (35.2)	3636 (48.7)	1000 (13.4)	204 (2.7)	22 (0.3)
Missing	3977	5100	2383	2037	360

Supplemental figure S1

Subtype-specific incidence of breast cancer by year at diagnosis in Sweden 2008-2019 – based on Multiple Imputed data

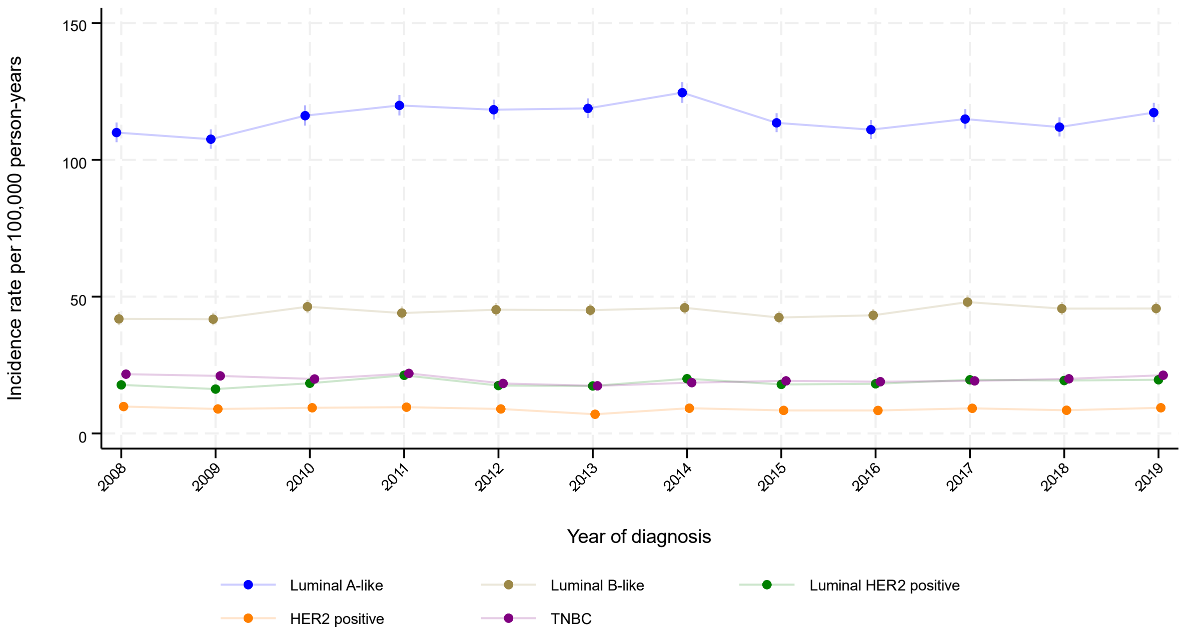

Supplemental figure S2

Overall breast cancer incidence by age at diagnosis in Sweden 2008-2019. Shaded area represents screening ages 40-74 years.

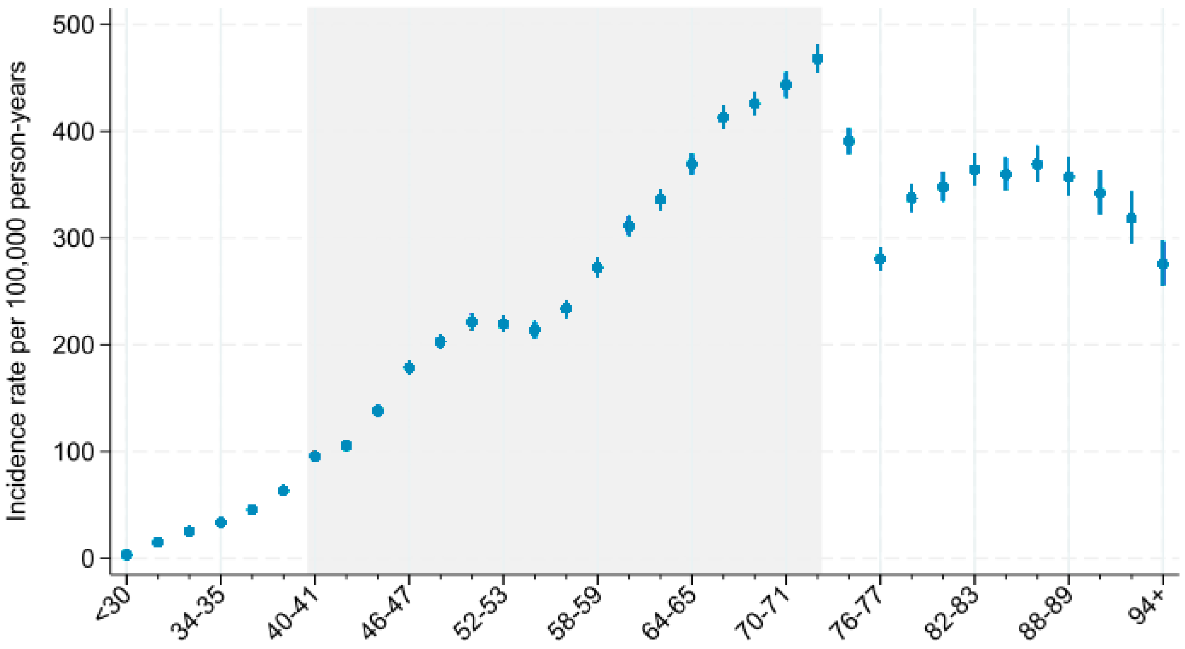

Supplemental figure S3

Estimated proportions of breast cancer subtypes by age at diagnosis in groupings that reflect the population-based screening programme in Sweden 2008-2019 – based on Multiple Imputed data

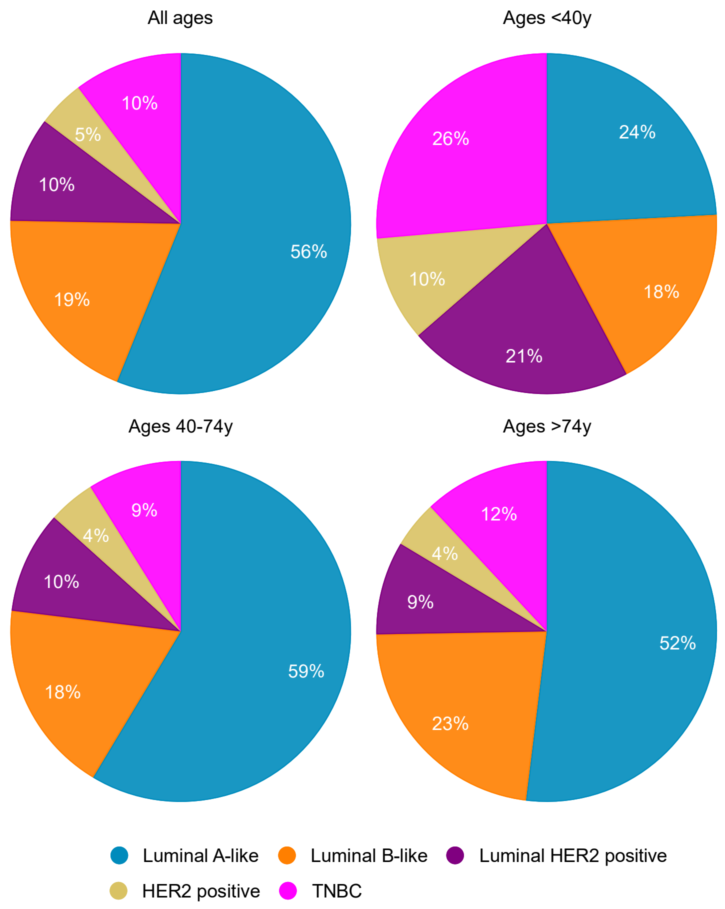

Supplemental figure S4

Estimated age-specific breast cancer incidence by surrogate subtypes in women up until age 45 in Sweden 2008-2019 – based on Multiple Imputed data. Shaded area represents screening ages 40-45 years.

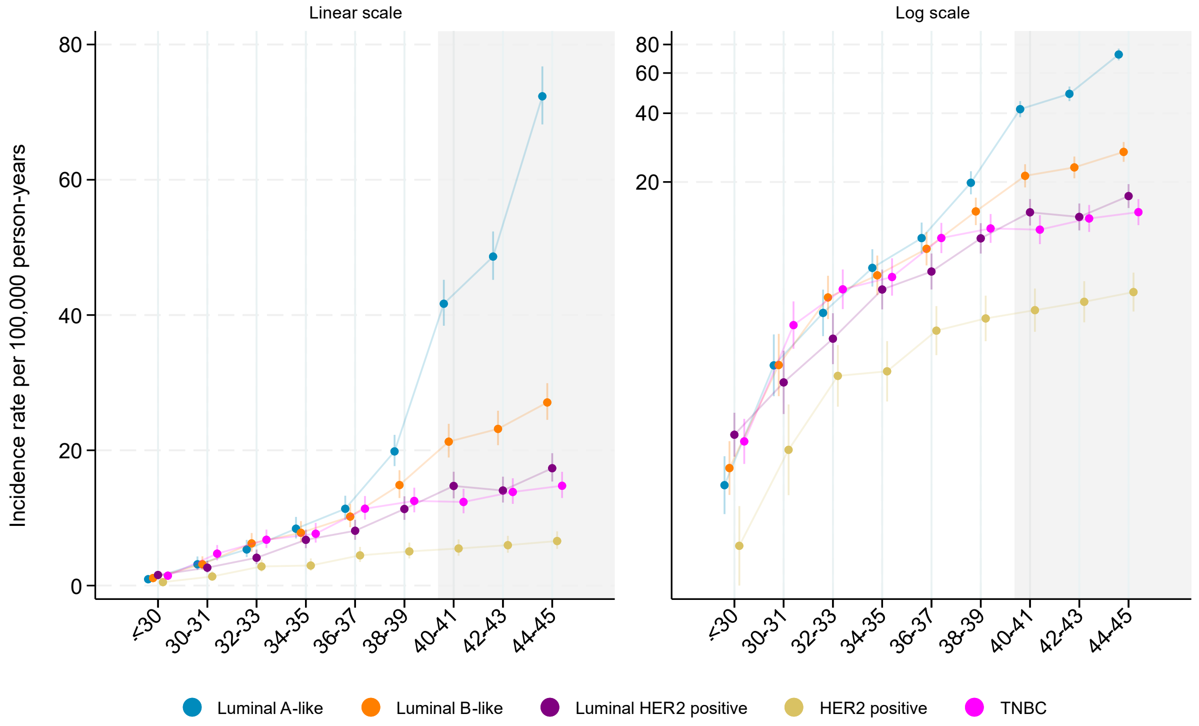

Supplemental figure S5

Incidence rate ratios (IRR) of stage-specific breast cancer incidence comparing complete case and imputation analyses for each breast cancer subtype.

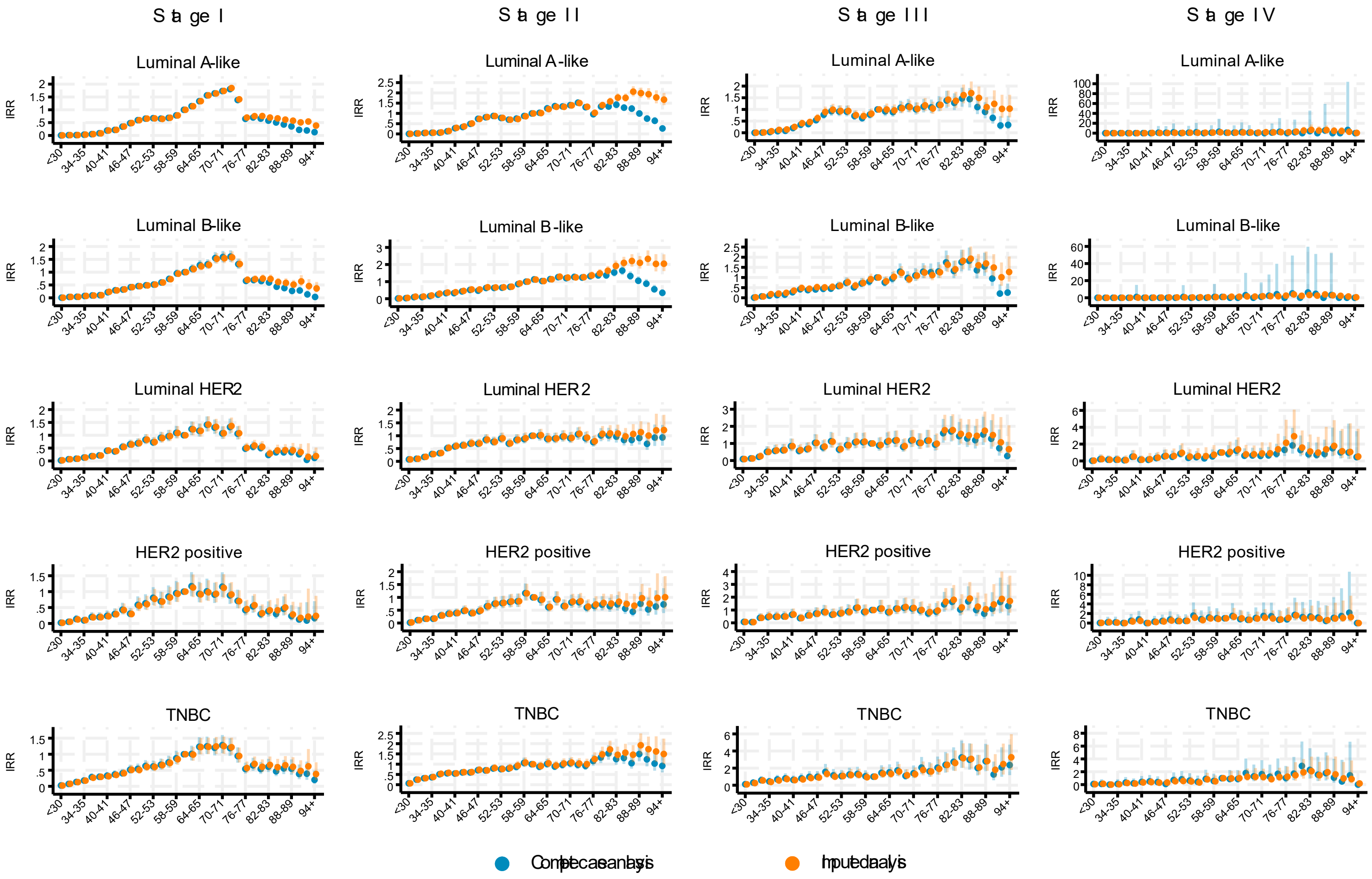

Supplemental figure S6

Estimated proportions of breast cancer stages by breast cancer subtypes in Sweden 2008-2019 – based on Multiple Imputed data.

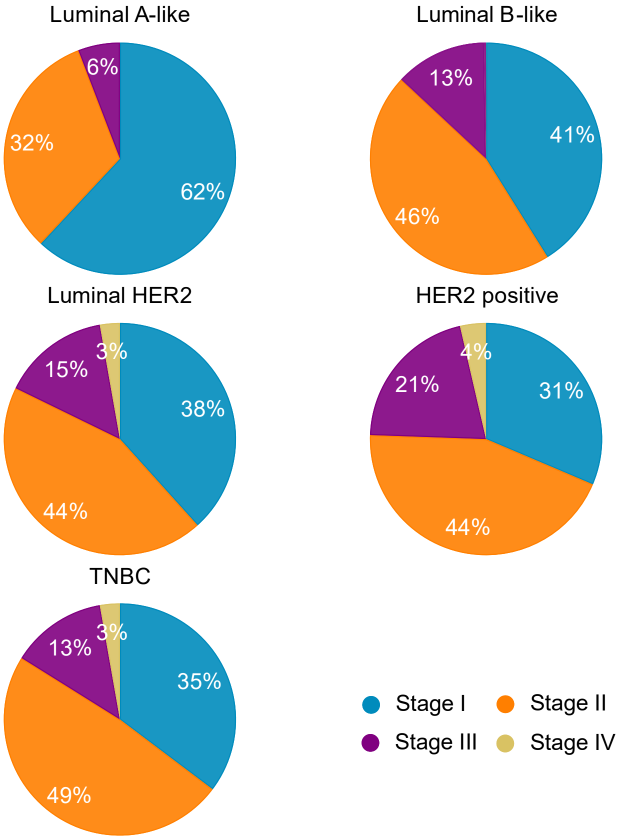

References

1. White IR, Royston P, Wood AM. Multiple imputation using chained equations: Issues and guidance for practice. Stat Med. 2011;30(4):377-399. doi:10.1002/sim.4067

2. Falcaro M, Nur U, Rachet B, Carpenter JR. Estimating excess hazard ratios and net survival when covariate data are missing: strategies for multiple imputation. Epidemiology. 2015;26(3):421-428. doi:10.1097/EDE.0000000000000283
